# Machine learning potential for interacting dislocations in the presence of free surfaces

**DOI:** 10.1038/s41598-022-07585-7

**Published:** 2022-03-08

**Authors:** Daniele Lanzoni, Fabrizio Rovaris, Francesco Montalenti

**Affiliations:** 1 Materials Science Department, University of Milano-Bicocca, Via R. Cozzi 55, 20125 Milan, Italy; 2 NOMATEN Centre of Excellence, National Centre for Nuclear Research, ul. A. Sołtana 7, Swierk, 05-400 Otwock, Poland

**Keywords:** Materials science, Theory and computation

## Abstract

Computing the total energy of a system of *N* interacting dislocations in the presence of arbitrary free surfaces is a difficult task, requiring Finite Element (FE) numerical calculations. Worst, high accuracy requires very fine meshes in the proximity of each dislocation core. Here we show that FE calculations can be conveniently replaced by a Machine Learning (ML) approach. After formulating the elastic problem in terms of one and two-body terms only, we use Sobolev training to obtain consistent information on both energy and forces, fitted using a feed-forward neural network (NN) architecture. As an example, we apply the proposed methodology to corrugated, heteroepitaxial semiconductor films, searching for the minimum-energy dislocation distributions by using Monte Carlo. Importantly, the presence of an interaction cutoff allows for the application of the method to systems of different sizes without the need to repeat training. Millions of energy evaluations are performed, a task which would have been impossible by brute-force FE calculations. Finally, we show how forces can be exploited in running 2D ML-based dislocation dynamics simulations.

## Introduction

Machine Learning (ML) methods have been recently exploited in a large number of applications in materials science^1–4^, solid-state physics^5^, molecular chemistry^6–10^, and crystallography^11^. The application of ML methods in these fields are allowing for data-intensive tasks that were previously considered inaccessible, such as the compositional search for material-discovery^2,4,12^ or the automated defect detection and classifications^13^, and are replacing conventional simulation techniques by allowing for better accuracy with faster computational time. In particular, the application of ML to the development of Force Fields (FFs) for Molecular Dynamics (MD) simulations^14–18^ have been proposed as a way to overcome the limitation of the classical modeling approaches. Established simulations procedures have relied up to now on performing very computationally demanding ab initio MD simulations, limited to a few hundreds of atoms, or on the exploitation of approximate empirical potentials. The large interest raised by ML techniques for the development of FF is due to the unprecedented trade-off between accuracy and computational speed allowed by ML^19^. The high level of flexibility provided by ML models such as Neural Networks (NN)^14,20,21^ and Gaussian Processes^16,22–24^ have been successfully exploited to faithfully reproduce first-principle atomistic calculations. This is achieved by evaluating large databases of atomistic configurations starting from ab-initio approaches such as Density Functional Theory (DFT)^17,18,25,26^ or Coupled Cluster^27^. This database is then used to train a ML model in predicting forces and energies for arbitrary atomistic configurations. The resulting FF is more computationally demanding than classical empirical potentials typically used in MD simulations of solids but it has been demonstrated that can be used to accurately reproduce ab-initio calculations with just a fraction of their computational cost^19^.

Here we show that a similar approach can be implemented also on the continuum scale for the modeling of dislocations within the linear elasticity framework. Describing the movement and arrangement of dislocations in a material is fundamental because these defects are the carrier of plasticity at the microscale. The mechanical properties of crystalline materials are dominated by their movement and interactions^28,29^. They are also commonly encountered during the fabrication of semiconductor devices in microelectronic as they can be easily generated in the relaxation process of thin films and heterostructures^30–33^. Interest in the application of ML for the modeling of dislocations has been recently raised^34^ and some successful applications of ML have been already demonstrated^35–37^ but these previous work focus more on reproducing complex collective behavior of dislocations, without putting emphasis on the details of the behavior of individual dislocations.

In our approach, however, we seek to develop a framework akin to the one used in atomistic FF evaluations in order to develop a ML model for high accuracy prediction of both energies and forces for simulations of dislocations. By exploiting a NN-based model we were able to predict the interaction between the defects, closely reproducing a properly-designed database of Finite Element (FE) calculations. We recall that the numerical evaluation of the dislocation–dislocation interaction is required whenever the problem involves the presence of complex free surface geometries^28,38^. In this kind of problems no analytical expressions for the stress/strain field of dislocations are available and thus the forces and energies produced by the defects need to be evaluated by solving the Partial Differential Equation (PDE) of mechanical equilibrium^39,40^. The inclusion of both energies and forces predictions in our approach represents a novel advancement with respect to previous works in this field. This is particularly relevant as this allow for the exploitation of our ML method both in minimum-search algorithms exploiting energy evaluations, both in Dislocation Dynamics approaches where the interaction forces between dislocations are required.

After separating the problem of interacting defects into convenient one and two-body terms, we used Sobolev training, in which both energies and forces are consistently learned by the NN^41,42^.

We then apply our ML approach to a relevant physical system, searching for the optimal arrangement of Misfit Dislocations (MDs) in a corrugated SiGe/Si heteroepitaxial film modeled in two dimensions (2D), for the sake of simplicity. Even so, the problem of finding the minimum energy dislocation distribution cannot be tackled by brute force, at least for large enough simulation cells (and assigned defect density), considering the proliferation of local energy minima^43^. The NN model developed here is thus applied as a high-throughput approach to a Monte Carlo (MC) minimum energy search at a fraction (approximately one order of magnitude less) of the computational cost of a single FE calculation (out of the more than $10^5$ required). This allowed us to statistically analyze the dislocation distributions, confirming existing experimental observations and theoretical predictions regarding this kind of systems.

## Methods

**Figure 1:**
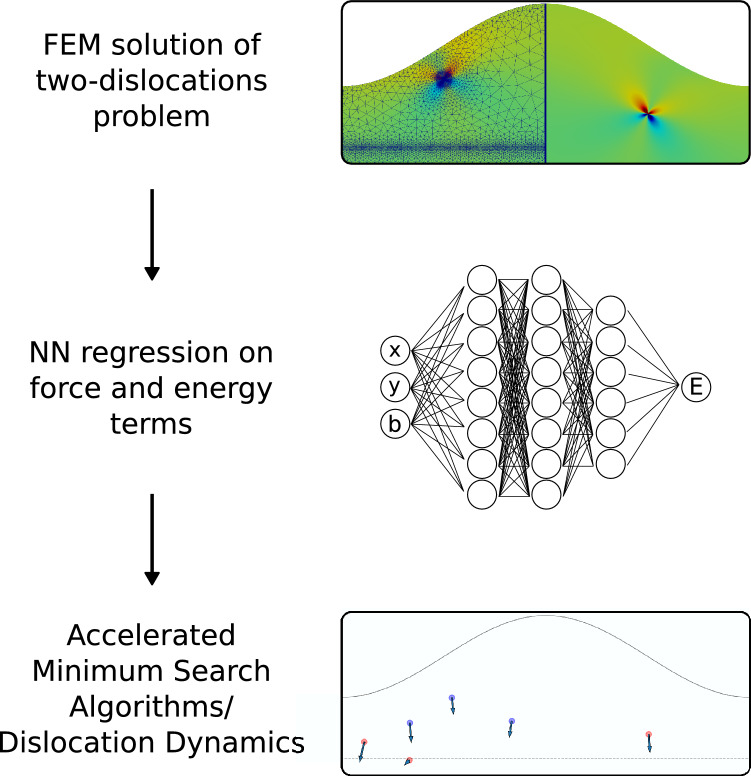
Sketch of the proposed method. A TS is constructed exploiting the interaction decomposition by numerically solving two-dislocation problems. Neural Networks are used to build predictive models of energies and forces of dislocation configurations. These are then used to search for low energy configurations or dislocation dynamics simulations orders of magnitude faster than by FE bruteforce evaluations.

The method presented in this Paper seeks to develop high accuracy predictions for forces and energies produced by an ensemble of *N* interacting dislocations near a free surface of generic geometry. In this section we will present a description of the general features of the method, as sketched in Fig. 1. As stated in the “Introduction”, our approach is inspired by the wide adoption of ML in the field of atomic FF calculations, we derive a similar method in the linear elasticity framework regarding dislocations. Following this analogy, first a high-accuracy evaluation of force/energy contributions is performed by means of FE calculations in order to obtain a trusted set of data, called the Training Set (TS). This is achieved by considering one and two body terms, exploiting the functional form of the dislocation–dislocation interaction as will be shown later in this section. The system is a thin heteroepitaxial film hosting dislocations, here modeled in two dimensions for the sake of simplicity. We considered a representative non-flat geometry for the free surface of the layer modeled by a sinusoidal perturbation as in the top inset of Fig. 1. Once the TS has been computed, we train a NN based on those data and test its performance on an independent set of data, the validation set. Finally, the trained NN can be used to perform high-throughput algorithms for minimum energy search or force-based physical models.

### Energy and forces decomposition

The energy of a system of *N* dislocations near an arbitrary surface with an arbitrary external stress field in a elastic body can be written as^44,45^:

1 $$\begin{aligned} E_{\text {tot}} = \sum _i^N V_i + \sum _i^N H_i + \frac{1}{2} \sum _i^N \sum _{j \ne i}^N W_{ij}, \end{aligned}$$

where *V*, *H* and *W* are, respectively, the dislocation self-energy, dislocation-external field interaction and the dislocation–dislocation interaction. In the following, term *H* will be referred as “dislocation-heteroepitaxial field interaction”, as this is the case we are interested in the current work. In the framework of linear elasticity this decomposition is exact. The terms under summation can be expressed in different ways. For example, self energy of a dislocation can be written as a function of the traction its own stress field exerts on the slip plane^45^. Another equivalent approach is to consider the expression of the elastic energy stored in a deformed body. This can be obtained by an integral over the whole material domain $\Omega$, as^44^:

2 $$\begin{aligned} V_i = \frac{1}{2} \int _\Omega \sigma _i(\vec {x}) : \varepsilon _i(\vec {x}) \text {d}\vec {x}, \end{aligned}$$

where $\sigma _i$ and $\varepsilon _i$ are the stress and strain fields generated by dislocation *i*, respectively. Similar results can be obtained for terms $H_i$ and $W_{ij}$. A complete derivation of these expressions can be found in the Supplementary Information of this Paper. For fixed boundary conditions and infinite straight dislocations, the only case we shall consider here, the value of $V_i$ and $H_i$ are functions of the dislocation position and Burgers vector only. Similarly, $W_{ij}$ depends only on the position and Burgers vector of dislocations *i* and *j*. The forces can be obtained by considering the gradient of Eq. (1). The force on the *i*th dislocation is:

3 $$\begin{aligned} \vec {F}_i = -\vec {\nabla }_i V_i - \vec {\nabla }_i H_i - \sum _{j \ne i}^N \vec {\nabla }_i W_{ij}, \end{aligned}$$

where $\vec {\nabla }_i$ is the gradient with respect to the position of dislocation *i*. This expression corresponds to the Peach-Koehler formula, well known in dislocation theory^44,45^:

4 $$\begin{aligned} \vec {F}_i = [\sigma (x_i,y_i) \cdot \vec {b}_i] \times {\hat{\xi }}_i, \end{aligned}$$

where $\sigma$ is the total external stress tensor acting on the position of dislocation *i*, $\vec {b}_i$ and ${\hat{\xi }}_i$ are the *i*th dislocation Burgers vector and dislocation line direction. Requiring the consistency between the two formulations, we can identify the following terms:

5 $$\begin{aligned} {\left\{ \begin{array}{ll} -\vec {\nabla }_i V_i = (\sigma _\text {self} \cdot \vec {b}_i) \times {\hat{\xi }}_i \\ {}-\vec {\nabla }_i H_i = (\sigma _\text {het} \cdot \vec {b}_i) \times {\hat{\xi }}_i \\ {}-\vec {\nabla }_i W_{ij} = (\sigma _j \cdot \vec {b}_i) \times {\hat{\xi }}_i \end{array}\right. } \end{aligned}$$

the terms $\sigma _\text {self}$, $\sigma _\text {het}$ and $\sigma _j$ indicating respectively the dislocation self stress, the heteroepitaxial stress field and the stress field generated by the *j*th dislocation at the position of dislocation *i*. We emphasize that self stress indicates here the stress field present at the dislocation core position as an effect of the free surface boundary conditions. Singular “bulk” terms, therefore, do not contribute to Peach–Koehler force^44,45^.

This energy and forces decomposition presents several advantages when building a database for the application of ML algorithms. There is a reduction in the complexity of models, leading to reduced overfitting problems, smaller memory requirements and faster evaluation times. A second advantage is that generalization to an arbitrary number of dislocations is a straightforward summation as in Eq. (1) and the database can be constructed considering only one and two body interactions.

### Data generation

As stated in the “Introduction”, analytical expressions for the stress/strain field of dislocations are available only in the simple case of dislocations in bulk systems or near a flat free surface^44,45^. All other non-trivial configurations require the numerical solution of the equilibrium PDE for an elastic continuum. In this work we numerically solved this equation by exploiting a MATLAB FE solver, like in Ref.^39^. In order to avoid divergences in dislocation cores we used the regularization approach presented in Ref.^46^ which also ensures consistency between the derivatives of the total elastic energy, Eq. (3), and the Peach–Koehler formula, Eq. (4). Locally-refined meshes were used in order to efficiently reproduce the high variations in the stress/strain field in the proximity of dislocation cores.

The system was modeled by considering the heteroepitaxial strain due to the lattice mismatch betweeen $\text {Si}_{0.75}\text {Ge}_{0.25}$ and Si(001) and the dislocations considered as infinite straight lines perpendicular to the system. Periodic boundary conditions were applied, similarly to Ref.^39^. Simulation cell was 1200 nm wide. The total stress/strain fields were evaluated by FE by setting the heteroepitaxial and dislocation strain fields as the initial condition for the mechanical equilibrium PDE with the help of the eigenstrain formalism (Refs.^39,40^).

In our work, we considered Burgers vectors only between the two possible relaxing orientations of $60^{\circ }$ dislocations. These are indeed the kind of defects that are normally nucleated in the Ge/Si(001) and SiGe/Si(001) system^30,31^. The sign of the in-plane (i.e. laying on the growth plane) component of the Burgers vector is fixed due to the need to relax the compressive misfit strain, while in a full 2D description only the sign of the out-of-plane component can vary. This reduces the choice for the Burgers vector to the two possibilities: $b_1 = b[\frac{1}{2}, \frac{1}{\sqrt{2}}, 0]$ and $b_2 = b[\frac{1}{2}, -\frac{1}{\sqrt{2}}, 0]$, with $b=0.3857~\text {nm}$.

As described in the Introduction, we focused on the effect of the free surface shape on the minimum-energy distribution of dislocations. Therefore, several TSs have been constructed by random sampling both positions and Burgers vectors of two-dislocation configurations for each different surface morphology. Initially, a TS with flat free surface have been generated using FE, even though an analytical solution is available in this particular case. This served as a baseline comparison between our method and an exact treatment. Next, TSs with perturbed surfaces were constructed. In particular, we chose a simple sinusoidal perturbation, used as a prototypical variation from the flat profile that integrates naturally with periodic boundary conditions. Periodicity of the perturbation was kept fixed to 600 nm (half the simulation cell), while oscillation amplitude *A* was varied. Each TS comprised approximately 12000 two-dislocation configurations. Periodicity and symmetries of the simulation cell has been exploited for TS augmentation.

Starting from the FE solution, strain/stress fields of dislocation, as well as $V_i$, $H_i$ and $W_{ij}$ values have been collected and the forces acting on dislocations have also been inserted in the TSs by exploiting Eq. (4). In this work, reported energies are in the units in which Young modulus for Si is unity.

### Model training and validation

The ML model used to learn the energy and force terms was a feed-forward, fully-connected NN. The choice of this approach over a Gaussian Process Regression comes from the constant computational costs with respect to the dimension of the TS, which can therefore be expanded without increasing evaluation times. The architecture chosen was the following: for one-body terms $H_i$ and $V_i$, the NNs had three hidden layers with 30, 20 and 20 neurons each, while for $W_{ij}$ the NN had four hidden layers with 40, 40, 40 and 20 neurons respectively. The activation functions chosen were hyperbolic tangents. No particular attempts in tuning the number of hidden units and other hyperparameters was performed, as our main concern was the feasibility of the proposed approach. NN implementation relied on the PyTorch framework^47^. The first training regression was made using a Mean Squared Error loss function on energy values. The functional to be minimized with respect to NN parameters $\theta _k$ reads like:

6 $$\begin{aligned} L(\{\theta _k\}) = \frac{1}{N_\text {TS}} \sum _i^{N_\text {TS}} [E(\vec {x_i}) - \hat{E}(\vec {x_i}| \{\theta _k\}) ]^2, \end{aligned}$$

where $N_\text {TS}$ is the number of elements in the TS, index *i* runs on TS elements, $\vec {x_i}$ represent the parameters defining the configuration (i.e. dislocation positions and Burgers vectors). In the following, fitting procedures using Eq. (6) will be referred as “Value training” (following Ref.^48^). Notation *E* indicates true values of the energy functions *V*, *H* and *W* as extracted by FE solutions, and $\hat{E}$ indicate the NN approximation.

**Figure 2:**
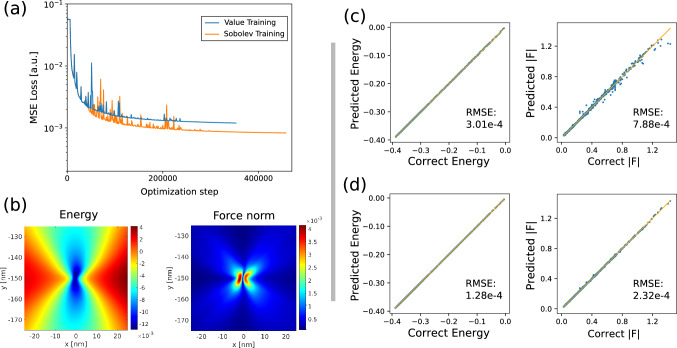
(**a**) Loss function calculated on the validation set during NN parameters optimization using Value (blue line) and Sobolev training (orange line). (**b**) Plot of energy and force near a dislocation as predicted from the NN model. Regression plots for the *H* term (both energies and forces) obtained from the model predictions on a validation set. A free surface with a perturbation of $60~\text {nm}$ amplitude was considered: value training (**c**) and Sobolev training (**d**).

As stated in the previous section, solving the mechanical equilibrium equation for a distribution of dislocations allows the collection of energy derivatives through Peach–Koehler forces. These can be used to enhance regression results through a procedure called Sobolev training^48^. Equation (6) can be modified by explicitly inserting derivatives of the NN output as, for example, in^49^:

7 $$\begin{aligned} \begin{aligned} L_\text {S}(\{\theta _k\}) = \frac{1}{N_\text {TS}} \sum _i^{N_\text {TS}} \{ [ E(\vec {x_i}) - \hat{E}(\vec {x_i} | \{\theta _k\}) ]^2 + \sum _l g_l [ F_l(\vec {x_i}) + \partial _l \hat{E}(\vec {x_i} | \{\theta _k\})]^2 \}, \end{aligned} \end{aligned}$$

where *l* is an index running on cartesian components of the *i*th dislocation position. $g_l$ is a weight parameter accounting for the relative importance of forces in regression. In our work we fixed its value so that relative errors in energies and forces contribute in the same way to the total loss. Notice that in Sobolev training, the forces are not an independent output of the NN, but are obtained by differentiation of the energies. This leads to a fully consistent model.

A comparison between Value and Sobolev has been performed on two NNs initialized with the same weights and hyperparameters using a validation set of configurations the models were not exposed during training. Training has continued until the relative decrease in the loss function was less than $10^{-6}$ in 1000 optimization steps. Convergence of the two different training approaches has been monitored by analyzing the evolution of the loss on a validation set versus the number of optimization steps. In order to have a fair comparison between Sobolev and Value training, Mean Squared Error Loss on the predicted energies only has been used for both algorithms. In Fig. 2a results are displayed for the $H_i$ term (dislocation-field interaction) for a sine-perturbed surface (amplitude 60 nm). It is clear that convergence is reached in both cases without overfitting. Such nice behavior is determined by the large number of examples present in the TS ($\approx 22,000$, considering data augmentation) with respect to the number of parameters in the networks ($\approx 1100$). The risk of overfitting is further reduced when using Sobolev training, as the information content for every data point is effectively augmented.

Regression plots are reported in Fig. 2c,d for Value and Sobolev training respectively. As can be clearly appreciated from the figure, Sobolev training performs better, especially in predicting forces. This is corroborated by the comparison between Root Mean Square Errors (RMSE), which shows a reduced error for both energy and forces, confirming that Sobolev training leads to equivalent or better predictions of both quantities. The energy and forces terms are also shown in the colormaps of Fig. 2b, as predicted by the trained NN model.

### Interaction cutoff

The method presented so far allows for a high-throughput simulation approach for dislocation in thin films but its application is limited to the simulation cell used to build the TS. A straightforward solution to this problem is the introduction of a cutoff radius beyond which dislocation-dislocation interactions can be considered null. This approach is justified by the presence of free surfaces, which ensures that dislocation fields decay more rapidly ($\propto r^{-2}$) than fields of bulk dislocations ($\propto r^{-1}$)^45^. Readers are addressed to the Supplementary Information file for more information regarding the choice of the cut-off scheme used and its performance.

## Results and discussion

In this section we present the results obtained by applying the model discussed and validated in “Methods”. We investigated a system consisting of *N* dislocations in a corrugated $\text {Si}_{0.75}\text {Ge}_{0.25}$/Si(001) film. In order to model a representative non-flat geometry we considered a sinusoidal perturbation of the heterolayer free surface, described by its amplitude *A*. The search for minimum-energy configurations for this kind of systems with several dislocations has been proven to be a difficult task due to the observed proliferation of local energy minima^43^. The absence of analytical solutions for the case of a non-flat free surface geometry would thus require expensive numerical evaluations for the total elastic energy of the system, making the brute-force search for the global energy minimum an unreachable task.

Furthermore, while often the minimum energy configuration is never achieved in real systems hosting dislocations, it has been shown that in semiconductor heteroepitaxy the lowest energy configurations can indeed provide valuable information on the relaxation process and even closely reproduced experimental results. A review of the main achievements in the field of heteroepitaxy can be found in the book by Hull^30^. Good agreements between theoretical predictions and observed dislocation distributions were found for dislocated heteroepitaxial islands, where the minimum energy criteria has been proved to be useful in determining the onset of plasticity^50,51^ or peculiar phenomena such as the cyclic growth of islands^39,52^. In thin films, ordered arrays of misfit dislocations have been ovbserved at the interface between the heteroepitaxial layer and the substrate^53,54^, confirming theoretical findings regarding the lowest energy distribution for non-graded layers^30,39,43^. Finally, in systems hosting few dislocations like the Vertical Heterostructures^33^, the onset of plasticity and the experimentally observed dislocation distributions have been observed to be in good agreement with the theoretical predicitons^40,55–57^.

These factors make the following analysis an ideal case for the application of the high-throughput model presented in this Paper. The minimum-energy search was here performed by a Monte Carlo algorithm exploiting the energy evaluations predicted by the trained NN discussed in “Methods”. Starting from a random dislocation configuration, at each simulation step a dislocation is selected and displaced. The new configuration is retained with probability $\exp {-\Delta E/k_BT}$, being $\Delta E$ the energy difference between initial and final configurations, $k_B$ Boltzmann constant and *T* the temperature of the system. A simulated annealing in which, after an initial minimization at absolute zero, temperature is progressively increased (up to  $1000~\text {K}$) and then decreased has been performed in order to reduce local minima trapping. After this first temperature ramp, a second energy minimization at absolute zero is performed.

**Figure 3:**
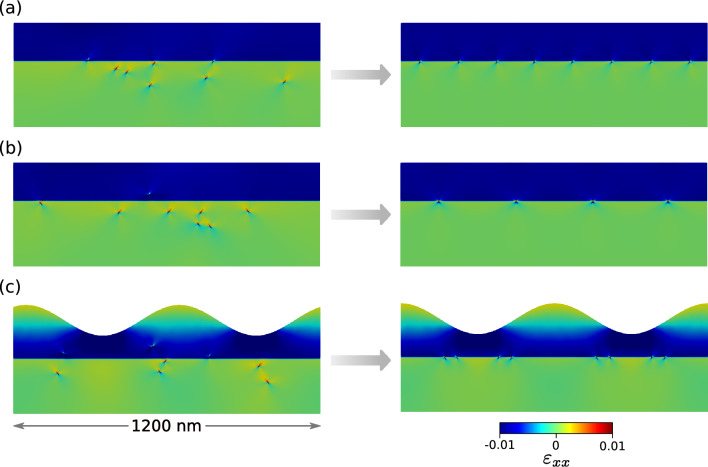
Example of low energy configurations obtained by minimum searches (right) starting from random initial configurations (left). Colormap indicates the xx component of strain field. As expected, regular arrays of $60^{\circ }$ (**a**) or edge dislocations (**b**) are observed. When the free surface is perturbed, edge dislocation formation is inhibited (**c**) and defects place below valleys in the surface profile.

The algorithm discussed above was applied to a system with $N=8$ dislocations. In order to validate the physical soundness of the results of our model we started by investigating a thin flat film where exact analytical expressions for the dislocation-dislocation interaction are available^58^ and the minimum-energy configurations are already known and well described in the literature^30,43^. As discussed in “Data generation”, we modeled two possible $60^{\circ }$ dislocations. For the scope of these simulations we considered two distributions of dislocations, one with all equal Burgers vector $\vec {b} = \vec {b_1}$ and the other with 50% of Burgers vectors $\vec {b_1}$ and the remaining $\vec {b_2}$. In the following we will refer to those distribution as “equal” and “alternated” Burgers vector, respectively. Results are shown in Fig. 3a,b for the equal and alternated distributions. In the case of alternated Burgers vector we allowed the reaction of two $60^{\circ }$ dislocations to form a single edge dislocation as expected by dislocation theory^28,44^. The resulting defect has a Burgers vector $\vec {b} = \vec {b_1} + \vec {b_2}$ and the reaction was allowed provided that the two original defects fell inside a typical cutoff distance $r_c = 5 \text {nm}$ during the simulated annealing. The final result shows, as expected, an ordered array of dislocations placed at the $\text {Si}_{0.75}\text {Ge}_{0.25}$/Si interface. For the case of dislocations with equal Burgers vectors, Fig. 3a, they form an array with all dislocations equally spaced and placed at the interface. On the other hand, for the case of alternated Burgers vector, each dislocation reacts with its complementary defect forming an edge dislocation. The result is an array of 4 edge dislocations equally spaced and placed at the $\text {Si}_{0.75}\text {Ge}_{0.25}$/Si interface, as shown in Fig. 3b. Furthermore, in Fig. 3c we present the results obtained by applying the Monte Carlo algorithm to a system with a free surface described by a sinusoidal perturbation of amplitude $A = 60~\text {nm}$. Here we can see that dislocations with alternated Burgers vector do not react anymore to form edge dislocations but place themselves in alternated fashion under the valley of the perturbation, directly showing the interplay between the surface morphology and the optimal dislocation positioning^39,59^. This results resembles the optimal positioning of $60^{\circ }$ dislocations in a heteroepitaxial island, providing optimal strain relaxation^52,60,61^. If the energies of the final configurations, as predicted from our NN functions, is compared with the correct energies evaluated by the FE solver, we observe a relative deviation in the order of 2‰. This confirm the accuracy of the predictions of our method, making the minimum energy search an ideal application of the presented approach.

**Figure 4:**
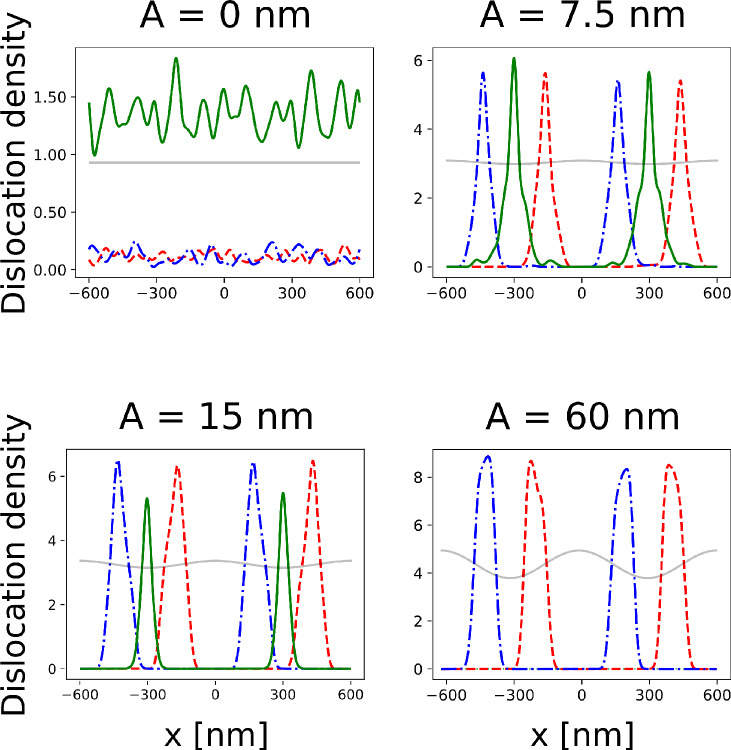
Dislocation density for different Burgers vectors as a function of the amplitude of the sinusoidal perturbation of the free surface (500 Monte Carlo searches each). Free surface profile is reported in transparency. As expected, for flat films (A = 0 nm) dislocations react and form edge dislocations (green solid line). Increasing the intensity of the perturbation, the density of $60^{\circ }$ dislocations (red dashed and red dot-dashed lines) increase and they tend to localize near valleys in the free surface profile (A = 7.5 nm and A = 15 nm). At the highest perturbation value (A = 60 nm) the formation of edge dislocations is completely prevented.

The interplay between the surface morphology and the optimal dislocation positioning has then been further analyzed. Thanks to the speedup provided by the ML model we performed several Monte Carlo minimization (500 for each considered configuration) starting from random initial dislocation distributions and different values for the amplitude of the sinusoidal perturbation *A*. In Fig. 4 we plot the dislocation densities obtained by averaging all the (smoothed) dislocation positions at the end of our MC minimization algorithm. The averaging is required in order to deal with the proliferation of local minima observed in these systems^43^. Despite the smoothing step the trend is clear: increasing the amplitude *A* produces a polarization in the positioning of the dislocations, depending on their Burgers vectors. At $A = 0$ most of the dislocation pairs react to form edge dislocations that are placed at the interface. This is expected from the observation of the lowest-energy configuration of Fig. 3b. In this case the dislocation densities of the $60^{\circ }$ dislocations (red dashed and red dot-dashed lines) are almost zero everywhere while the density of edge dislocations (green solid line) is nearly constant because all the positions at the interface are energetically equivalent. When the amplitude increases a lower number of edge dislocation is formed, corresponding to a higher probability of finding $60^{\circ }$ dislocations aligned with the valleys of the perturbation. At the maximum *A* value considered, $A=60~\text {nm}$, as in Fig 3c, a clear polarization in the positioning of dislocation with respect to their Burgers vector is observed. In this case no edge dislocations are formed in all the MC simulations attempted. The density plot thus reflects the net polarization observed in the lowest-energy configuration found, already reported in Fig 3c, where all the $60^{\circ }$ dislocations are placed on the lateral sides of the valleys of the perturbation, depending on their Burgers vector.

**Figure 5:**
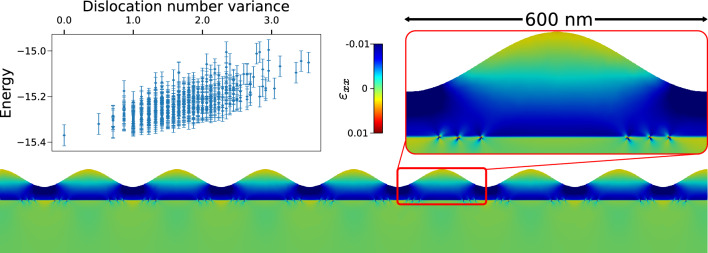
Minimum energy configuration obtained on a cell 4800 nm long using cutoff. Colormap shows *xx* component of the strain field. Left inset shows the energy of found local minima as a function of the variance of the number of dislocations below each valley in the free surface. Error bars give indicative errors in energy (approximately 3‰ of energy values). Right inset shows zooms in the vicinity of a single period of oscillation in the free surface. Dislocation separation by Burgers vector can be clearly observed.

Furthermore, in order to demonstrate an application of our approach to simulation cells larger than the one used to generate the TS we performed additional simulations exploiting the cutoff scheme described in “Interaction cutoff”. A large-scale MC minimum energy search was performed considering a simulation cell four times larger ($4800~\text {nm}$) than the one described above and used for the generation of the TS. In Fig. 5 we report the result of the MC minimization, obtained again starting with all the dislocation randomly placed in the whole simulation cell. The energy as a function of the variance in the number of dislocations for each periodicity of the free-surface perturbation is reported in the left inset. The independent variable in the plot has been chosen as a descriptor of the symmetry of the configuration: a value of 0 means that dislocations are evenly distributed along all the valleys of the simulation cell. Error-bars correspond to 3‰ of the the energy value, the total relative error between the lowest-found NN and FE-evaluated energies. As reported above (Fig. 3c) the lowest energy configuration is associated with an ordered distribution of dislocations with the defects aligned along the two sides of the perturbation valleys, depending on their Burgers vectors. This indicates that the error induced by the use of the cutoff scheme is not critical for minimum energy searches. The bottom part of Fig 5 shows the lowest energy configuration found. The trend of the energy distribution and the high symmetry of the configuration suggests this is a reasonable candidate for the global energy minimum of the system. Therefore, the restriction of the validity of the trained ML model to a specific geometry when building the database is partially overcome by the cutoff scheme here proposed.

**Figure 6:**
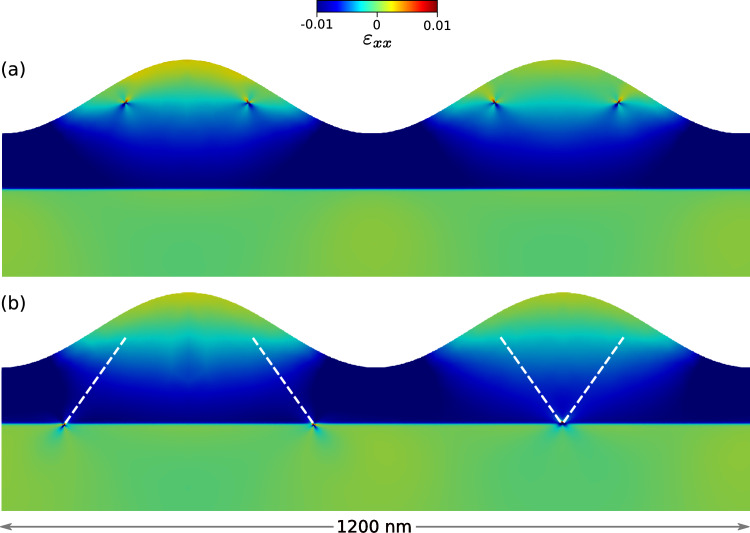
Initial (**a**) and final (**b**) configuration in a 2D Dislocation Dynamics simulation obtained using forces from NN functions. Dislocation paths on their glide planes are reported as dashed white lines. A sessile edge dislocation is formed in the middle of the simulation cell.

Finally, while the search for energy minima is an interesting application of our method per-se, in most dislocation simulations the motion of the defects is bound to prescribed crystallographic directions called Glide Planes (GPs), in order to mimick the behavior of these defects in real systems. This is exploited in approaches like the Dislocation Dynamics (DD) where the simulations predict the time evolution of a given initial dislocation microstructure. While in 3D this procedure requires complex tracking of dislocation line topology, in 2D DD reduces to a constrained motion of point-like particles moving under the influence of the Peach–Koehler force. As reported in “Model training and validation”, the NN model trained with the Soboelev training also provide reliable force predictions. It is therefore possible to run DD simulations with surface-corrected interactions. One of such application is reported in Fig. 6. Initial (Fig. 6a) and final (Fig. 6b) configurations are shown for a pair of dislocations. On the left part of the Figure it is possible to observe the motion of two dislocation with different Burgers vector that follow different GPs (highlighted by the white dashed lines). Their motion ends at the intersection between the GPs and the $\text {Si}_{0.75}\text {Ge}_{0.25}$/Si interface. The final result shows the two defects aligned at the two sides of the perturbation as seen in the previously discussed MC results. However, by switching the Burgers vector of the two defects the result changes completely, as can be seen in the right part of the Figure. This happens because the defects are still restricted to move on their GPs and thus they glide towards the center, reacting with each other and forming a single edge dislocation. This result, as discussed before, do not represent the optimal positioning of the defects in this system but show a typical outcome when considering the constrained motion of this crystallographic defects.

## Conclusions

In this paper we presented a ML model for high-accuracy predictions of forces and energies for dislocations simulation. The scheme of our method was inspired by the adoption of ML in the field of atomistic FF evaluations. We trained a NN model with the help of a properly-evaluated database of FE calculations and exploited the Sobolev training to enhance the predictive ability of our model. Furthermore, the separation of the problem into one and two-body terms allowed by the functional form of the dislocation-dislocation interaction allowed us to train a very high accuracy NN model with a reasonable-sized TS.

The resulting NN was then applied for producing fast energy and forces evaluations in algorithms such as MC minimum energy search or DD in a corrugated $\text {Si}_{0.75}\text {Ge}_{0.25}$/Si thin film. The obtained results were in agreement with several existing observations regarding these systems but a complete statistical investigation was made possible only by the speedup provided by ML. The total wall time of a MC energy minimization exploiting the ML model was about 1 min, orders of magnitude faster than the brute-force FE calculations would have required (more than 2 years keeping the same accuracy used to build the TS). This demonstrates how new possible ways of investigating this systems are allowed by the enhanced predictive ability of such a high-throughput model.

Extension of the present model to 3D systems requires a major effort in order to deal with the additional complexities raised by the full description of the dislocation lines, but a first extension considering straight dislocations has already been envisaged.

## Supplementary Information

Supplementary Information.

## Data Availability

The authors declare that all data supporting the findings of this study are available within the Paper and its Supplementary Information file. Further information are available upon reasonable request.
