# Author Correction: Machine learning potential for interacting dislocations in the presence of free surfaces

**DOI:** 10.1038/s41598-023-38180-z

**Published:** 2023-07-11

**Authors:** Daniele Lanzoni, Fabrizio Rovaris, Francesco Montalenti

**Affiliations:** 1 Materials Science Department, University of Milano-Bicocca, Via R. Cozzi 55, 20125 Milan, Italy; 2 NOMATEN Centre of Excellence, National Centre for Nuclear Research, ul. A. Sołtana 7, Swierk, 05-400 Otwock, Poland

Correction to: *Scientific Reports* 10.1038/s41598-022-07585-7, published online 08 March 2022

The Funding section in the original version of this Article was omitted. The Funding section now reads:

“F.R. acknowledges support from the European Union Horizon 2020 research and innovation programme (Grant Agreement No. 857470) and from the European Regional Development Fund via the Foundation for Polish Science International Research Agenda PLUS programme (Grant No. MAB PLUS/2018/8).”

The original Article has been corrected.

