# Supplementary material for: Machine learning potential for interacting dislocations in the presence of free surfaces
Source: Sci Rep. 2022 Mar 8;12:3760. doi: 10.1038/s41598-022-07585-7 (PMC8904841; doi:10.1038/s41598-022-07585-7)
Supplement: Supplementary file 1 — Supplementary Information. [file 41598_2022_7585_MOESM1_ESM.pdf]

Machine Learning Potential for Interacting Dislocations in the Presence of Free Surfaces

Daniele Lanzoni[1], Fabrizio Rovaris[1,2], and Francesco Montalenti[1]

[1]Materials Science Department, University of Milano-Bicocca, Via R. Cozzi 55, I-20125 Milano, Italy
[2]NOMATEN Centre of Excellence, National Centre for Nuclear Research, ul. A. Sołtana 7, 05-400 Swierk/Otwock, Poland

1 Energy decomposition

We prove that the energy of an arbitrary configuration of N dislocations in an heteroepitaxial film can be written in the form:

$$E_{\text{tot}} = \sum_i^N V_i + \sum_i^N H_i + \frac{1}{2}\sum_i^N \sum_{j\neq i}^N W_{ij} \tag{S1}$$

From the linear theory of elasticity, the energy of a (traction-free) deformed body Ω can be expressed as [1, 2]:

$$E_{\text{tot}} = \frac{1}{2}\int_\Omega \sigma_{\text{tot}} : \varepsilon_{\text{tot}} \mathrm{d}\vec{x} \tag{S2}$$

where σ_{tot} and ε_{tot} are the total stress and strain fields in the material. Expanding these terms with respect to heteroepitaxial field and dislocation contributions, Eq. (S2) can be rephrased as:

$$E_{\text{tot}} = \frac{1}{2}\int_\Omega \Big(\sigma_{\text{het}} + \sum_i^N \sigma_i\Big) : \Big(\varepsilon_{\text{het}} + \sum_j^N \varepsilon_j\Big)\mathrm{d}\vec{x} \tag{S3}$$

where "het" subscript indicates fields generate by the heteroepitaxial mismatch and i and j refer to dislocations. Notice that we decomposed the stress/strain terms in heteroepitaxial and dislocation contributions for consistency with the specific application in the Paper. Eq. (S3) and the following statements still hold if an arbitrary external stress/strain field replaces the heteroepitaxial field and arbitrary stress/strain inducing defects replace dislocations.

Distributing the tensor product :, the total energy reads

$$
\begin{aligned}
E_{\mathrm{tot}} =& \frac{1}{2}\int_{\Omega} \sigma_{\mathrm{het}} : \varepsilon_{\mathrm{het}} \mathrm{d}\vec{x} + \\
& \sum_{i}^{N} \int_{\Omega} \sigma_{\mathrm{het}} : \varepsilon_{i} \mathrm{d}\vec{x} + \\
& \frac{1}{2}\sum_{i}^{N} \int_{\Omega} \sigma_{i} : \varepsilon_{i} \mathrm{d}\vec{x} + \\
& \frac{1}{2}\sum_{i}^{N}\sum_{j\neq i}^{N} \int_{\Omega} \sigma_{i} : \varepsilon_{j} \mathrm{d}\vec{x}
\end{aligned}
\tag{S4}
$$

Physical interpretation of terms in Eq. (S4) is as follows. The first term represents the elastic energy stored in a non-dislocated film. In our work we disregarded its value as we were not interested in comparing the total energy of different film geometries, but it could be easily inserted in further developments of our method. The second and third terms represent the dislocation-heteroepitaxial film interaction and the so-called dislocation self-energy, i.e. the distortion energy associated with the dislocation presence by its own existence. It can be clearly seen that, once surface morphology is fixed, the value of these terms only depends on the position and Burgers vector of dislocation i. The last term is the dislocation-dislocation interaction energy. Comparison between Eq. (S1) and Eq. (S4) yields:

$$
\begin{aligned}
H_i &= \int_{\Omega} \sigma_{\mathrm{het}} : \varepsilon_{i} \mathrm{d}\vec{x} \\
V_i &= \frac{1}{2}\int_{\Omega} \sigma_{i} : \varepsilon_{i} \mathrm{d}\vec{x} \\
W_{ij} &= \int_{\Omega} \sigma_{i} : \varepsilon_{j} \mathrm{d}\vec{x}
\end{aligned}
\tag{S5}
$$

2 Cutoff radius and interaction convergence

In order to extend our results to simulation cells bigger than those present in the TSs we exploited a cutoff scheme setting to zero the dislocation-dislocation interactions when their horizontal separation was greater than 600 nm. This procedure introduce an approximation error in energy evaluations. In order to quantify this error, we plotted the relative difference in energy at the cutoff value for different dislocation array (and simulation cell) periodicity in Figure S1. Dislocations were placed 150 nm below a flat free surface, as this profile allows an analytical expression of stress fields, and energies have been obtained by Peach-Koehler force integration. The reference value E_{∞} is that of interaction between isolated dislocations without periodic images in an semi-infinite solid with flat free surface.

The periodicity chosen in our work is 1200 nm, which ensures a relative error of less than 1%. In order to reduce by another order of magnitude, a periodicity greater than 2000 nm would be required, corresponding to a cutoff radius of more than 1000 nm.

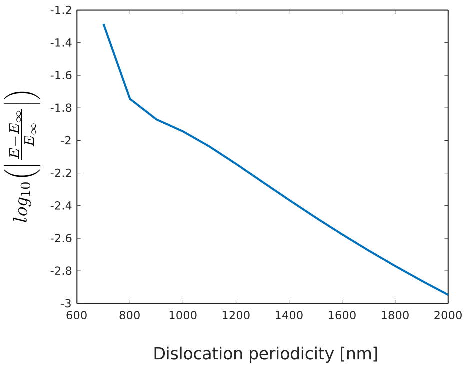

Figure S1: Relative difference in dislocation interaction energies for dislocation 600 nm apart and 150 nm below a flat free surface as a function of the cell periodicity. The reference value E_∞ is that of an isolated dislocation without periodic images.

3 Regression plots

In this Section we report for completeness all the regression plots for the Soboelev training as discussed in Section 2.3 of the Paper. In Figure S2 we show regression plots for all the terms of Eq. (S5) regarding both Energies and Forces for the case of a perturbed free surface with $A = 60$ nm.

References

[1] J.P. Hirth and J. Lothe. *Theory of Dislocations*. Krieger Publishing Company, 1982. ISBN 9780894646171. URL `https://books.google.it/books?id=LFZGAAAAYAAJ`.

[2] Robin W Lardner. *Mathematical theory of dislocations and fracture*. University of Toronto Press, 1974.

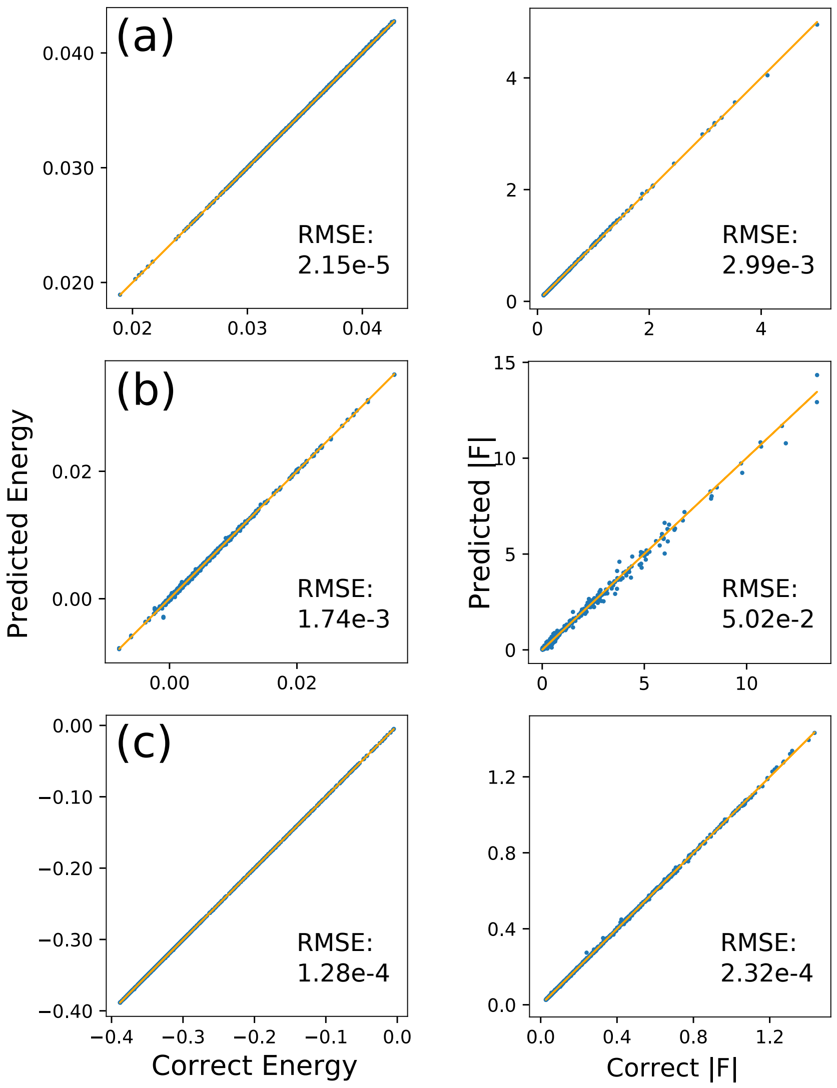

Figure S2: Regression plots for V_i (a), W_{ij} (b) and H_i (c) for both energies (left) and forces (right), as obtained from Sobolev training. Terms are relative to a perturbed free surface with amplitude $A = 60$ nm.
